# Dynamic Adjust of Non‐Radiative and Radiative Attenuation of AIE Molecules Reinforces NIR‐II Imaging Mediated Photothermal Therapy and Immunotherapy

**DOI:** 10.1002/advs.202104793

**Published:** 2022-01-22

**Authors:** Zhenjie Wang, Ling Yu, Yuehua Wang, Chenlu Wang, Qingchun Mu, Xiaojing Liu, Meng Yu, Kang‐Nan Wang, Guangyu Yao, Zhiqiang Yu

**Affiliations:** ^1^ The People's Hospital of Gaozhou Maoming 525200 P. R. China; ^2^ Second Clinical College Guangzhou University of Chinese Medicine Guangzhou 510006 P. R. China; ^3^ AMI Key laboratory of Chinese Medicine in Guangzhou Guangdong Provincial Hospital of Chinese Medicine Guangzhou 510120 P. R. China; ^4^ Cancer Center Integrated Hospital of Traditional Chinese Medicine Southern Medical University Guangzhou 510315 P. R. China; ^5^ MOE Key Laboratory for Analytical Science of Food Safety and Biology College of Chemistry Fuzhou University Fuzhou 350108 P. R. China; ^6^ Guangdong Provincial Key Laboratory of New Drug Screening School of Pharmaceutical Sciences Southern Medical University No. 1023, South Shatai Road Guangzhou 510515 P. R. China; ^7^ Shunde Hospital Southern Medical University (The First People's Hospital of Shunde) Foshan 528308 P. R. China; ^8^ Breast Center Department of General Surgery Nanfang Hospital Southern Medical University Guangzhou 510515 P. R. China

**Keywords:** AIE, CD39‐CD73‐A2AR, immunogenic cell death, NIR‐II, photothermal therapy

## Abstract

Due to the aggregation‐caused quenching effect and near‐infrared I poor penetration capabilities of common fluorescent molecules, their applications in visualized imaging and photoactivated treatment are limited. Therefore, new near‐infrared II (NIR‐II) molecule (named TST), which had the abilities of aggregation‐induced emission (AIE) and photothermal therapy are synthesized. Moreover, in order to further improve its fluorescent yield and therapeutic effect, camptothecin prodrug (CPT‐S‐PEG) and novel immune checkpoint inhibitor AZD4635 are used to co‐assemble with TST into nanoparticles for drug delivery. On account of the strong interaction of camptothecin and TST, the intramolecular rotation of TST is limited, thereby inhibiting non‐radiation attenuation and promoting fluorescence generation when the nanoparticles are intact. As nanoparticles uptake by cancer cells, redox sensitive CPT‐S‐PEG is degraded and the nanoparticles disintegrate. The released TST enhances non‐radiative attenuation and expedites photothermal conversion because of the removal of the constraint of camptothecin. Furthermore, photothermal therapy induces immunogenic cell death of cancer cells and releases abundant ATP into the tumor microenvironment to recruit immune cells. However, superfluous ATP is converted into immunosuppressive adenosine through the CD39‐CD73‐A2AR pathway. The AZD4635 released by photothermal disintegration of the nanoparticles just blocks this pathway timely, achieving favorable synergistic effect of photothermal therapy, chemotherapy, and immunotherapy.

## Graphical abstract

By dynamically adjusting the radiative attenuation and non‐radiative attenuation of aggregation‐induced emission molecules, the effect of enhancing near infrared region II imaging and photothermal therapy can be achieved simultaneously. Moreover, photothermal therapy cause immunogenic cell death. The released camptothecin inhibits DNA topoisomerase to play a role in chemotherapy and the released AZD4635 restrains CD39‐CD73‐A2AR pathway to enhance immunotherapy.

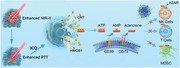

## 1 Introduction

The development of optical agents for tumor phototheranostics has recently attracted great interest because they allow real‐time molecular diagnosis and parallel phototriggered therapy.^[^ ^1^, ^2^, ^3^, ^4^ ^]^ It requires these agents to simultaneously emitter infrared fluorescence to imaging and has photodynamic or photothermal capabilities. And photoactivated photothermal therapy (PTT) has been established as safe model for tumor ablation in many cancer indications.^[^ ^5^, ^6^, ^7^, ^8^ ^]^ Photothermal therapy can cause local thermal damage in the tumor area. However, the common photothermal agents such as indocyanine green (ICG) and IR780 have aggregation‐caused quenching (ACQ) properties owing to intermolecular interactions (*π*–*π* stacking and other non‐radiative decays), thus tremendously limit their application in cancer phototheranostics.^[^ ^9^, ^10^, ^11^, ^12^ ^]^ Since the ideal system for emission and heat production is still limited by the difficulty of blocking strong *π*–*π* stacking of molecule in nanoparticles, there is an urgent need to develop effective method to prepare advanced phototheranostic nanoparticles that can produce both high fluorescence and heat for cancer theranostics. The problem is perfectly solved by aggregation‐induced emission (AIE) agents and has received more and more attention in recent years.^[^ ^13^, ^14^, ^15^ ^]^ AIE agents are often non‐emissive in solution due to the depletion of the excited state energy through non‐radiative relaxation by intramolecular motion. But when AIE molecules aggregated, such relaxation from the lowest excited singlet state (S_1_) to the ground state (S_0_) is largely restricted due to the steric hindrance, leading to the energy of S_1_ going through the fluorescence pathway to S_0_.^[^ ^16^, ^17^, ^18^ ^]^ Xinggui Gu et al reported nanoparticles made of corannulene‐PEG loaded AIE molecule, which strongly suppressed non‐radiative attenuation and promoted fluorescence and ROS production due to further limiting the intramolecular rotation of AIE molecule.^[^ ^19^ ^]^ But this only promoted fluorescence production, not photothermal generation, because photothermal conversion mainly relied on non‐radiative attenuation of AIE agents. Therefore, it is necessary to develop a smart nanodrug delivery system that can be dynamically adjusted non‐radiative and radiative attenuation of AIE molecules.

Photothermal treatment can induce cancer cells to immunogenic cell death (ICD), which is typically released calreticulin (CRT), high mobility group box 1 (HMGB1) and ATP to extracellular, thereby recruiting immune cells.^[^ ^20^, ^21^, ^22^ ^]^ However, ATP turns into adenosine by CD39 and CD73 ectoenzymes participation, which binds to the A2A receptor (A2AR) of immune cells to suppress their activity. To further improve the efficacy of tumor immunotherapy, many inhibitors of the CD39‐CD73‐A2AR pathway have been developed.^[^ ^23^, ^24^, ^25^, ^26^ ^]^ AZD4635 is an A2AR antagonist with high affinity and specificity for A2AR. AZD4635 as a single agent and in combination with durvalumab (anti‐PD‐L1 antibody) in patients with solid malignancies which is currently in clinical trials.^[^ ^27^, ^28^ ^]^ It proves that AZD4635 has good safety and therapeutic effect.

**Scheme 1:**
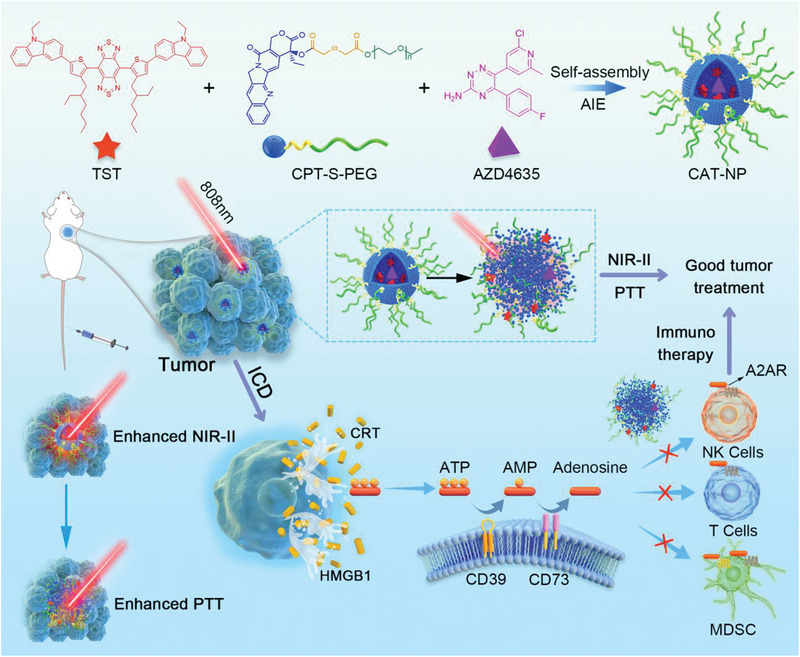
The TST, CPT‐S‐PEG, and AZD4635 were co‐assembled into nanoparticles (CAT‐NP) through nanoprecipitation method. CAT‐NP can be passively targeted to tumor tissues through EPR effect after intravenous injection of Balb/c mice. The CAT‐NP showed enhanced NIR‐II imaging due to AIE property when the nanoparticles were intact and enhanced PTT when nanoparticles were disintegrated. PTT induced the ICD of cancer cells thus released large amounts of CRT, HMGB1, and ATP into the tumor microenvironment. But ATP was converted into immunosuppressant adenosine through the CD39‐CD73‐A2AR pathway. The AZD4635 released by the disintegrated nanoparticles can inhibit the binding of adenosine to the A2AR receptor of immune cells, thus enhancing the immunotherapy effect of tumors.

Here, we synthesized a new AIE molecule with both near‐infrared II (NIR‐II) imaging and photothermal properties, which named TST. And NIR‐II (1000–1700 nm) had deeper tissue penetration (up to 15 mm), which resulted in higher imaging resolution than NIR‐I (700–900 nm).^[^ ^29^, ^30^, ^31^, ^32^ ^]^ Camptothecin (CPT) was a cytotoxic quinoline alkaloid that has lots of conjugated double bond. CPT and PEG were linked by esterification to form amphiphilic compounds with redox‐sensitive single‐sulfur linker in the middle (CPT‐S‐PEG).^[^ ^33^, ^34^, ^35^, ^36^ ^]^ CPT‐S‐PEG, TST, and AZD4635 were co‐assembled into nanoparticles by nanoprecipitation method. Due to the strong interaction between CPT and TST, the non‐radiative attenuation of TST was restrained, resulting in stronger NIR‐II fluorescence when the nanoparticles were intact. As nanoparticles uptake by cancer cells, redox sensitive CPT‐S‐PEG was degraded and the nanoparticles disintegrated. The released TST enhanced non‐radiative attenuation and accelerated photothermal conversion due to get rid of the restriction of CPT. Moreover, as the temperature of the tumor site was raised by photothermal action, tumor cells underwent ICD. The released CPT inhibited DNA topoisomerase to play a role in chemotherapy and the released AZD4635 restrained CD39‐CD73‐A2AR pathway to enhance immunotherapy. In conclusion, as shown in **Scheme** **1**, we have successfully designed a smart nanodelivery system that is capable of photothermal killing of tumor cells while conducting surgical navigation by NIR‐II imaging, and successfully realized the synergy of chemotherapy and immunotherapy.

## 2 Results and Discussions

### 2.1 Characterization of TST Nanoparticles

**Figure 1:**
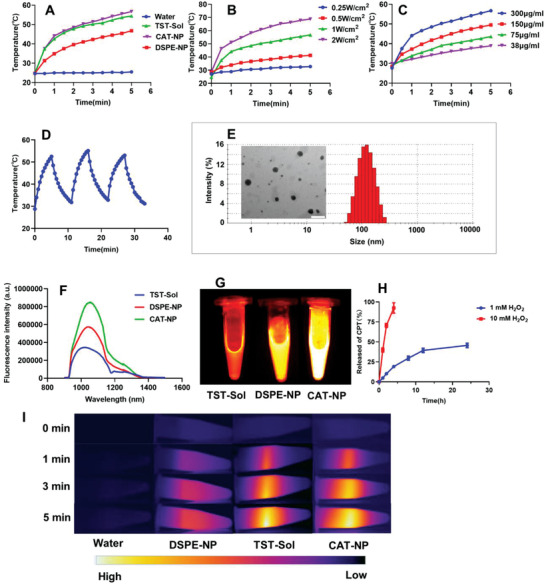
The characterization of TST nanoparticles. A) The temperature change of water, TST‐Sol, DSPE‐NP, and CAT‐NP under 808 nm laser irradiation at 1 W cm^−2^. B) The temperature change of CAT‐NP under different power of 808 nm laser irradiation. C) The temperature change of CAT‐NP with different concentrations under 808 nm laser irradiation at 1 W cm^−2^. D) The temperature change of CAT‐NP during three consecutive 808 nm laser irradiation and cooling cycles. E) The nanoparticle size measured by dynamic light scattering (DLS) and transmission electron microscope (TEM) images of CAT‐NP, scale bar: 200 nm. F) Emission fluorescence intensity curves of TST‐Sol, DSPE‐NP, and CAT‐NP at the same concentration after excitation with 808 nm laser. G) The NIR‐II images of TST‐Sol, DSPE‐NP, and CAT‐NP at the same concentration after excitation with 808 nm laser. H) The CPT release profile of C‐NP in 1 mm H_2_O_2_ and 10 mm H_2_O_2_ (*n* = 3). I) Photothermal images of water, TST‐Sol, DSPE‐NP, and CAT‐NP irradiated by 808 nm laser at 1 W cm^−2^.

TST has been successfully synthesized, and its synthesis method and characterization are described in Figures S1–S4, Supporting Information. And CPT‐S‐PEG was successfully synthesized by esterification reaction via EDCI/DMAP as catalyst (Figures S5–S6, Supporting Information). Then five kinds of nanoparticles were successfully prepared through nanoprecipitation method, including C‐NP (CPT‐S‐PEG 12 mg mL^−1^), CA‐NP (CPT‐S‐PEG 12 mg mL^−1^ and AZD4635 0.1 mg mL^−1^), CT‐NP (CPT‐S‐PEG 12 mg mL^−1^ and TST 0.3 mg mL^−1^), DSPE‐NP (DSPE‐PEG 12 mg mL^−1^, AZD4635 0.1 mg mL^−1^, and TST 0.3 mg mL^−1^), CAT‐NP (CPT‐S‐PEG 12 mg mL^−1^, AZD4635 0.1 mg mL^−1^, and TST 0.3 mg mL^−1^), and TST‐Sol (TST 0.3 mg mL^−1^, tetrahydrofuran solution). The photothermal performance of the different nanoparticles was investigated subsequently. After irradiating different solutions with 808 nm laser (1 W cm^−2^), the photothermal heating curve of CAT‐NP in the environment of 10 mm H_2_O_2_ (simulate the high oxidation environment in cancer cells) was almost consistent with TST‐Sol, and the highest temperature can reach 56.7 °C. However, the heating efficiency of DSPE‐NP was much lower than CAT‐NP and TST‐Sol, with maximum of 46.8 °C. Besides the temperature of water had remained constant at about 25 °C. The low photothermal efficiency of DSPE‐NP may be due to the fact that TST in the nanoparticles was in the state of aggregation, and the strong intermolecular interaction limited the intramolecular rotation and stretching vibration of TST, and inhibited non‐radiative energy attenuation, thus reducing the photothermal conversion efficiency. However, redox sensitive CPT‐S‐PEG can be degraded by high concentrations of H_2_O_2_ in the environment, resulting in the disintegration of the nanoparticles and releasing TST. The released TST relieved the inhibition of non‐radiative attenuation, thus the heating curve of CAT‐NP was basically consistent with that of TST‐Sol (**Figure** **1**A,I). Figure 1B showed that the photothermal performance of CAT‐NP was laser power dependent. As the laser power was raised from 0.25 to 2 W cm^−2^, the final temperature of the nanoparticles system was raised from 32.7 to 68.8 °C after continuous laser irradiation for 5 min. Meanwhile, the laser power was fixed to 1 W cm^−2^ to irradiate different concentrations of nanoparticles, and the results showed that the photothermal properties of the CAT‐NP depended on its concentration. That is the higher nanoparticles concentration, the higher photothermal efficiency (Figure 1C). In addition, the photothermal reproducibility of CAT‐NP was verified by measurement of three consecutive heating and cooling cycles (Figure 1D). As shown in Figure 1E, CAT‐NP was spherical and the particle size was uniform about 120 nm. The zeta potential of CAT‐NP was about −35 mV. Fluorescence spectra of mixtures with different proportion of tetrahydrofuran and water upon emission wavelength at 900 and 1500 nm were obtained to study the AIE property of TST. As shown in Figure S7, Supporting Information, the TST was almost non‐emissive in tetrahydrofuran but enhanced fluorescence after being introduced in a mixture of tetrahydrofuran/water as the water content increased. The UV–vis–NIR absorption spectra of TST‐Sol was shown in Figure S8, Supporting Information, and there were four absorption peaks at 241, 306, 347, and 700 nm, respectively. The fluorescence intensity of different nanoparticles was scanned with the same power of 808 nm laser excitation, and it can be seen from Figure 1F, CAT‐NP had the highest fluorescence yield, compared to TST‐Sol and DSPE‐NP. And the strongest emission wavelength was 1050 nm. It was also proved by the NIR‐II photograph in Figure 1G. The fluorescence quantum yield of TST‐Sol was only 0.40% and CAT‐NP was 15.32% determined by integrating sphere method. This manifested that TST molecules really had the ability of imaging NIR‐II. Moreover, the imaging effect of the nanoparticles was better than that of the solution, and the CAT‐NP imaging capacity was superior to DSPE‐NP. The reason for this result is that TST was a molecule with AIE function, and the CPT molecules were strongly interacting with TST, limiting the intramolecular rotation of TST, thus strongly inhibited non‐radiation attenuation and promoting fluorescence production. Then simulate the high hydrogen peroxide oxidation environment of tumor cells, and investigate the capacity of CPT‐S‐PEG single‐sulfur bond cleaved to release the CPT. The results showed that the release rate of CPT depended on H_2_O_2_ concentration in environment (Figure 1H). In high hydrogen peroxide environment, CPT can be released almost completely (92.4%) within 4 h, while CPT was only released 45.6% in low hydrogen peroxide environment in 24 h. It testified the oxidation sensitivity of CPT‐S‐PEG. The stability of CAT‐NP was satisfactory and the data was shown in Figure S9, Supporting Information. Physicochemical characterization of TST nanoparticles showed that CAT‐NP had excellent imaging properties in AIE and NIR‐II, and outstanding synergistic effects of photothermal and chemotherapy.

### 2.2 In Vitro Evaluation of Antitumor Efficacy

**Figure 2:**
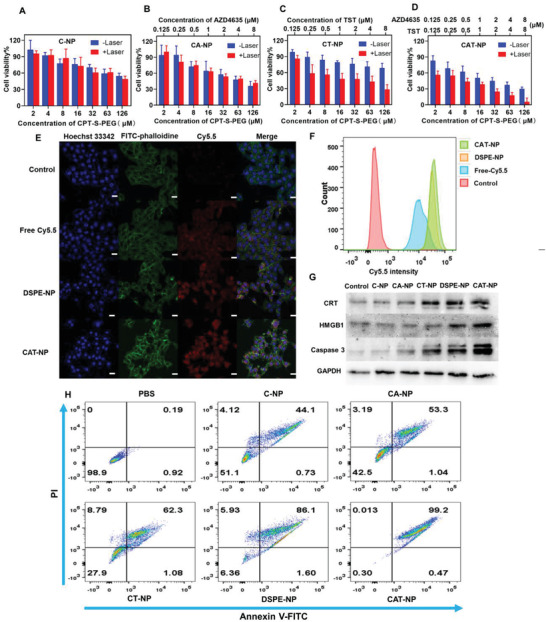
Cellular uptake and antitumor capacity of nanoparticles in vitro. Cytotoxicity of A) C‐NP, B) CA‐NP, C) CT‐NP, and D) CAT‐NP with or without laser irradiation (*n* = 6). E) The cellular uptake of free Cy5.5, DSPE‐NP, and CPT‐NP obtained by confocal laser scanning microscopy (Blue: Hoechst 33342‐stained nuclei, Green: FITC‐phalloidine stained cytoskeleton, Red: Cy5.5, scale bar: 20 µm). F) The cellular uptake of free Cy5.5, DSPE‐NP, and CPT‐NP obtained by flow cytometry. G) Western blotting of different nanoparticles treated 4T1 cells. H) The 4T1 cells apoptosis after treated with different nanoparticles measured by flow cytometry.

To further explore the antitumor effect of TST nanoparticles, a series of preliminary studies on 4T1 cell line were conducted. We first studied the cytotoxicity of four nanoparticles by MTT assay with or without 808 nm laser irradiation as control. The results showed that the cytotoxicity of four nanoparticles was concentration‐dependent, that is, the higher the concentration of nanoparticles, the stronger the cytotoxicity. The difference was that there were no TST‐loaded nanoparticles (C‐NP and CA‐NP), laser irradiation or not had little effect on their cytotoxicity. However, TST‐loaded nanoparticles (CT‐NP and CAT‐NP) showed stronger cytotoxicity after laser irradiation, especially CAT‐NP, which showed the strongest killing effect on tumor cells after laser irradiation (**Figure** **2**A‐D). Cell uptake of the nanoparticles was subsequently studied by flow cytometry and confocal laser scanning microscopy. As shown in Figure 2E, the blue channel was the Hoechst 33342‐marked nuclei, the green channel was phalloidine labeled cell skeleton, the red channel was lipophilic Cy5.5 and its red strongness represents the capacity of cell intake Cy5.5. After the same time of co‐incubation, the cell uptake efficiency of the nanoparticles group was higher than that of the free drug group, which might be because the small and uniform size of nanoparticles was more conducive to endocytosis, but the poor solubility of lipophilic free Cy5.5 in cell culture medium hindered its endocytosis. And the Figure 2F manifested the same result. Then the immunogenic cell death and apoptosis of different nanoparticles were studied via western blotting method. As shown in Figure 2G, CRT and HMGB1 were the signature proteins of ICD and caspase 3 are the marker protein of apoptosis. The results demonstrated that CAT‐NP caused the most severe ICD and apoptosis thus revealed that it had the best antitumor efficacy. Besides the experiment of apoptosis detection kit also proved it (Figure 2H). 4T1 cells were co‐incubated with different nanoparticles for 12 h, then each group irradiated with 808 nm laser respectively. After that, the cells were further cultured for 12 h and then double‐stained with Annexin V‐FITC and PI subsequently detected by flow cytometry. The results showed that CAT‐NP caused 99.7% of cell apoptosis, which indicated it had the strongest ability to kill cancer cells, compared with C‐NP 44.8%, CA‐NP 54.3%, CT‐NP 63.4%, and DSPE‐NP 87.7% of cell apoptosis.

### 2.3 In Vivo Imaging of TST Nanoparticles

**Figure 3:**
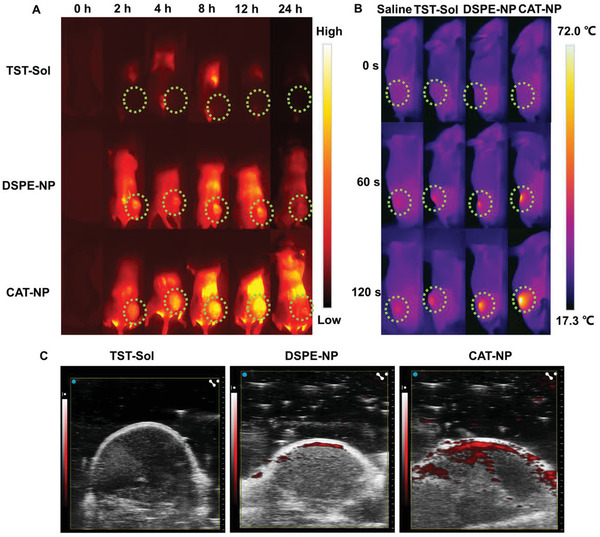
Study on the imaging properties of TST nanoparticles in vivo: A) NIR‐II image and dotted line circled the tumor, B) photothermal image, C) photoacoustic image.

In order to further explore the in vivo imaging performance of TST nanoparticles, when the tumor of 4T1 tumor‐bearing mice grew to 100–200 mm^3^, NIR‐II imaging, photothermal imaging and photoacoustic imaging were performed according to the specified time points after different preparations of TST were injected into the mice tail vein. NIR‐II image can be seen from **Figure** **3**A that the nanoparticles groups (DSPE‐NP and CAT‐NP) can passively target to the tumor due to enhanced permeability and retention (EPR) effect, while TST‐Sol cannot be enriched in the tumor site. Moreover, the TST‐Sol did not have long circulation effect in vivo, it accumulated maximum in the liver at 8 h, and then it was eliminated by the body metabolism. However, due to the presence of PEG, the nanoparticles groups had long circulation effect in vivo, with the largest accumulation in the tumor site at 12 h and cleared by system metabolism 24 h later. It is inspiring that the NIR‐II fluorescence intensity of CAT‐NP was stronger than that of DSPE‐NP in vivo. This testified that CAT‐NP still maintained high fluorescence yield in vivo, which was consistent with in vitro experiments. As shown in Figure 3B, CAT‐NP also displayed the optimal photothermal capability, which the maximum temperature of xenograft tumor rose to 72 °C after two minutes of 808 nm laser irradiation at power of 1 W cm^−2^. Not only that, CAT‐NP manifested excellent photoacoustic imaging as well (Figure 3C). All these suggested that CAT‐NP continued its splendid performance in vivo, due to the dynamic adjust of non‐radiative and radiative attenuation of AIE molecules.

### 2.4 Gene Sequencing of Different Nanoparticles Treated 4T1 Cells

**Figure 4:**
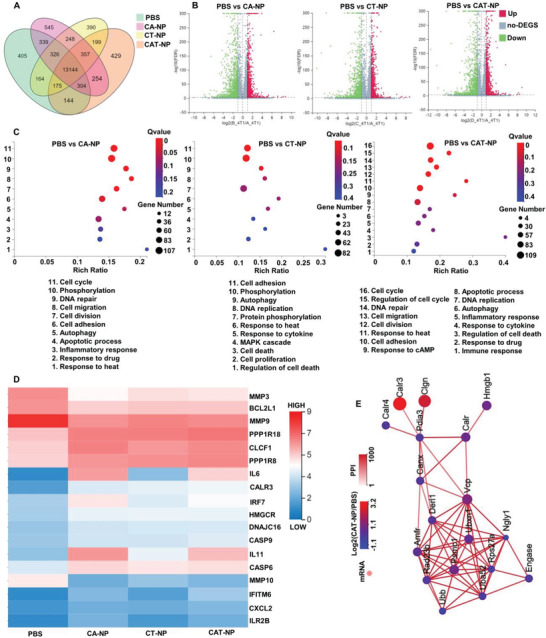
RNA sequencing of 4T1 cells after treatment with different nanoparticles. A) The VENN graph represented gene expression relationship between each group. B) Differential gene volcano map represented the number of up‐regulated or down‐regulated gene expressions between PBS and different nanoparticle groups. C) The enrichment analysis of Gene Ontology (GO) between different samples. D) Differential gene clustering heat map of different samples. E) The graph of protein–protein interaction (PPI) network compared with PBS and CAT‐NP group.

To further investigate the potential biological mechanism of the synergistic treatment of CPT, AZD4635, and TST in cancer cells, the whole genome RNA expression sequencing (RNA‐seq) in 4T1 cells exposed to various treatments was conducted. Afterwards, the Gene Ontology (GO)/Kyoto Encyclopedia of Genes and Genomes (KEGG) enrichment analysis was performed to obtain biological process and pathway differences between different samples. The gene expression relationship between each group was exhibited by the VENN graph (**Figure** **4**A). Among the 17 423 examined genes, there were 545, 390, and 429 gene transcripts uniquely upregulated in 4T1 cells treated by CA‐NP, CT‐NP, and CAT‐NP respectively. As shown in the volcano plots (Figure 4B), there were 1063 genes were upregulated while 1025 genes were downregulated in CAT‐NP compared to PBS group, which were much more significant of gene expression than that of CA‐NP (964 genes and 939 genes) and CT‐NP (914 genes and 852 genes). And based on Figure 4C, multiple signaling pathways were significantly affected by the nanoparticles we prepared, such as inflammatory response, apoptotic process, autophagy, cell migration, response to heat, and so on. It was worth noting that CAT‐NP group had the most influence on related signal pathway genes, for instance regulation of cell death, response to cAMP, response to cytokine and immune response. The up‐regulation or down‐regulation of related gene expression can be seen more clearly from the differential gene cluster heat map (Figure 4D). For example, the MMP3, MMP9, and BCL2L1 transcription of CAT‐NP group were down‐regulated upon inflammatory stimuli and apoptosis. The transcription of PPP1R8, CXCL2, CLCF1, CASP9, HMGCR, IL6, and IL11 were up‐regulated since CAT‐NP caused the most severe apoptosis and immunogenic cell death compared with PBS, CA‐NP, and CT‐NP. The graph of protein–protein interaction network showed ICD related genes of CAT‐NP group such as HMGB1, CLGN, CALR3, CALR, and VCP were up‐regulated compared to PBS group, which proved that CAT‐NP can induce ICD more effectively.

### 2.5 In Vivo Evaluation of Anticancer Efficacy

**Figure 5:**
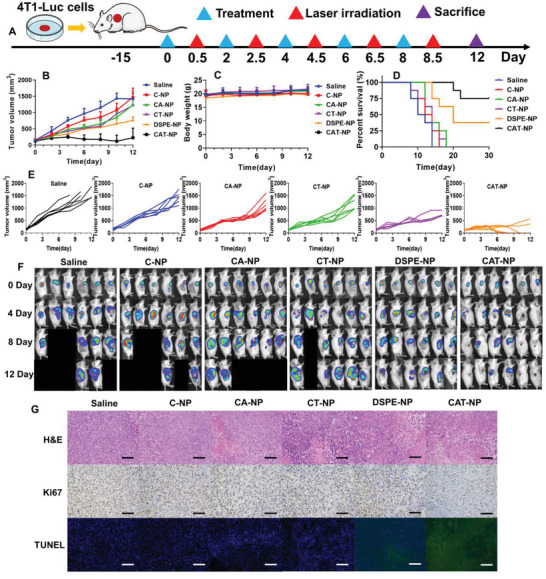
Study on antitumor efficacy of different nanoparticles in vivo. A) Schematic diagram of treatment plan. B) The overall growth curve of tumor in 4T1‐Luc bearing mice. C) The curve of body weight in mice. D) The survival curve of tumor‐bearing mice. E) The tumor growth curves of each group respectively. F) The small animal living image after intravenous injection of d‐luciferin at 0, 4, 8, 12 days. A black background without a mouse means the mouse is dead. G) The H&E, Ki67, and TUNEL stained tumor pathology section, scale bar: 100 µm.

To further investigate the antitumor effect of TST nanoparticles in vivo, 4T1‐Luciferase (4T1‐Luc) cells were inoculated subcutaneously under the armpit of Balb/c mice. When the tumor reached 100 mm^3^, the mice were treated with different nanoparticles injected into the tail vein. According to the previous in vivo imaging study of nanoparticles, these nanoparticles accumulated the most in the tumor site at 12 h after intravenous injection, so the mice subcutaneous tumors were irradiated with 808 nm laser at 12 h after the beginning of treatment. The detailed experimental scheme was shown in **Figure** **5**A. And Figure 5B,E were graphs of tumor growth curve in mice, revealing that CAT‐NP still showed the best in vivo antitumor effect, with tumor volume of 225 ± 203 mm^3^ after 12 days of treatment compared with DSPE (756 ± 108 mm^3^), CT‐NP (1215 ± 374 mm^3^), CA‐NP (1233 ± 418 mm^3^), and C‐NP (1469 ± 279 mm^3^). As shown in Figure 5C and Figure S10, Supporting Information, all the nanopreparations had satisfactory safety in vivo, no weight loss due to systemic toxicity, and no abnormalities were found in pathological sections of heart, liver, spleen, lung, and kidney. The liver and kidney function indexes of ALT, AST, BUN, and CR were determined, which again indicated that the nanoparticles were safe in vivo (Figure S11, Supporting Information). According to the survival curve of mice (Figure 5D), except for the DSPE‐NP and CAT‐NP groups, mice in other groups died within 18 days after the beginning of treatment. However, CAT‐NP group had 75% survival rate after 30 days of treatment, compared with 37.5% survival rate for DSPE‐NP group. Since the tumor model was inoculated with 4T1‐Luc cells, the tumor size of the mice could be directly observed by small animal living image system after the intravenous injection of d‐luciferin substrate. As shown in Figure 5F, tumor area was directly observed on day 0, day 4, day 8, and day 12 after treatment. The results showed that CAT‐NP was the most effective treatment. At day 12, four mice could not see any bioluminescence at the tumor site, demonstrating that CAT‐NP had killed overwhelming majority of the cancer cells. Next, H&E, TUNEL, and Ki67 immunohistochemical staining were performed on the pathological sections of tumor tissue (Figure 5G). Both H&E and TUNEL results suggested that CAT‐NP caused the most apoptosis and necrosis of cancer cells. H&E diagram showed that the tumor cells of CAT‐NP group sparsely spaced and had the largest area of necrosis. TUNEL diagram showed that CAT‐NP group had the highest desoxyribose uridine triphosphate labeled green fluorescence, manifesting the most apoptosis of cancer cells. Ki67 staining represented cell proliferation activity, and the more brown cells in the picture meant the stronger cell proliferation ability. As can be seen from Figure 5G, the brown part in CAT‐NP group was the least, which proved that CAT‐NP can effectively weaken the proliferation activity of cancer cells.

### 2.6 Investigation of Immunotherapy Effect of Different Nanoparticles In Vivo

**Figure 6:**
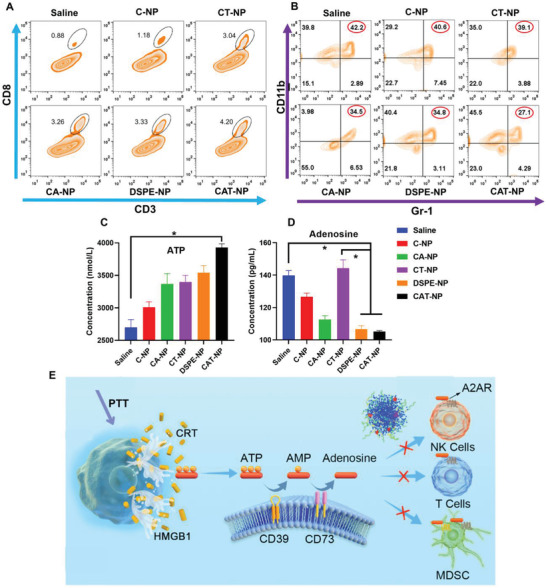
Investigation of immunotherapy effect of different nanoparticles in vivo. A) Cytotoxic T lymphocytes (CTL) in tumor sites detected by flow cytometry. B) Myeloid derived suppressor cell (MDSC) in tumor sites measured by flow cytometry. The content of C) ATP and D) adenosine in tumor microenvironment was determined by ELISA (*n* = 6). E) Schematic diagram of AZD4635 affected the CD39‐CD73‐A2AR pathway.

Because AZD4635 inhibits adenosine production, which activates tumor immune pathways, then tumor‐site immune cells were examined by flow cytometry. As shown in **Figure** **6**A, CD3 was a marker protein of total T lymphocytes, while CD8 was a marker of cytotoxic T lymphocytes (CTL). The circles in the image meant CTL enriched in tumor site and CAT‐NP group attracted the largest number of CTL to the tumor site (4.2%), compared to Saline group (0.8%). Similarly, CD11b was a marker of total macrophage, while Gr‐1 was marker of myeloid derived suppressor cell (MDSC). MDSC was the precursor of dendritic cells, macrophages and granulocytes, which had the ability to significantly inhibit the immune cell response. In Figure 6B, the cell population positive for both CD11b and Gr‐1 was MDSC, whose proportion in the whole cell has been circled in red. The proportion of MDSC in CAT‐NP was the smallest (27.1%), compared with the Saline group (42.2%). It revealed that CAT‐NP maximally inhibited the generation of MDSC and most effectively activated the response ability of immune cells. CD49b was the marker of NK cells, so CD49b positive cell mass were NK cells. As shown in Figure S12, Supporting Information, the CAT‐NP group recruited the hugest number of NK cells, up to 22.7% compared with 11.7% in the Saline group. Finally, ATP and adenosine levels in the tumor microenvironment were determined by enzyme linked immunosorbent assay (ELISA), and the results showed that photothermal therapy induced ICD of cancer cells, thus releasing more ATP into extracellular matrix to recruit immune cells. However, ATP was converted to adenosine via the CD39‐CD73‐A2AR pathway, which inhibited immune cell activity (Figure 6C). As shown in Figure 6D, CAT‐NP produced the largest amount of ATP due to the optimal photothermal treatment, but the adenosine content in the tumor microenvironment did not increase, furthermore it was the lowest of all groups. This was due to the role of AZD4635 in the nanosystem, which inhibited the conversion of ATP to adenosine. This also explained why CAT‐NP group recruited the most CTL and NK cells and inhibited the generation of MDSC to the greatest extent. It demonstrated that CAT‐NP showed the optimal synergistic effect of photothermal therapy, chemotherapy, and immunotherapy (**Figure 6E**).

## 3 Conclusion

In conclusion, in order to solve the problem that the current commercial fluorescent dyes are prone to ACQ and shallow fluorescence penetration when delivered in the body, we synthesized a novel NIR‐II molecule with both AIE and photothermal functions. These allow TST not only to visualize navigational surgery, but also to kill cancer cells through photothermal effect. In order to further improve the fluorescence yield and heat generation of TST, we designed CPT‐S‐PEG and AZD4635 to co‐assemble with TST into nanoparticles for intelligent drug delivery. Due to the strong interaction of CPT and TST, the intramolecular rotation of TST was limited, thereby strongly inhibiting non‐radiation attenuation and promoting fluorescence generation when the nanoparticles were intact. Whereas redox sensitive CPT‐S‐PEG was degraded and the nanoparticles disintegrated in tumor cells with high concentration of H_2_O_2_. The released TST enhanced non‐radiative attenuation and expedited photothermal conversion because of removing the constraint of CPT. Moreover, photothermal therapy induced ICD of cancer cell, which releases large amounts of ATP into the tumor microenvironment, thereby recruiting immune cells to kill cancer cells. However, superfluous ATP converted to adenosine via CD39‐CD73‐A2AR pathway, which inhibited the activity of immune cells. At this time, AZD4635 released by the photothermal disintegration of the nanoparticles just blocked this pathway, achieving excellent synergistic effect of photothermal therapy, chemotherapy and immunotherapy.

## 4 Experimental Section

### Materials

CPT was obtained from Meryer Co., Ltd., 3,3'‐Thiodipropionic acid, 1‐ethyl‐3(3‐dimethylpropylamine) carbodiimide (EDCI) and 4‐Dimethylaminopyridine (DMAP) were purchased from Macklin Co., Ltd., Methoxypolyethylene glycol (PEG2000) and Hoechst 33 342 were obtained from Aladdin Biochemical Technology Co., Ltd. DSPE‐PEG2000 was purchased from AVT (Shanghai) Pharmaceutical Tech Co., Ltd. AZD4635 was obtained from MedChemExpress (MCE). Cy5.5 (Cyanine5.5)‐carboxylic acid was purchased from Nanjing Goyoo Biotech Co.,Ltd. 96‐well, 12‐well, 6‐well plate were obtained from NEST Biotechnology. MTT and FITC‐phalloidine were purchased from Solarbio life sciences. Annexin V‐FITC/PI Apoptosis Detection Kit and d‐Luciferin sodium salt were obtained from Yeasen Biotech Co., Ltd. Mouse Adenosine and ATP ELISA Kit were purchased from Shanghai Jianglai Biotechnology Co., Ltd. The antibodies of CRT, HMGB1 and Caspase 3 were purchased from Abcam. The antibody of GAPDH and HRP labeled sheep anti‐rabbit secondary antibody were obtained from BIOSS.

The synthesis and characterization of TST and CPT‐S‐PEG can be found in the Supporting Information.

### Preparation of Co‐Assembly of Nanoparticles

CPT‐S‐PEG 12 mg, AZD4635 0.1 mg, and TST 0.3 mg were dissolved in 400 µL tetrahydrofuran, then they were slow dropped to the rapidly stirred 1 mL distilled water to make the size of nanoparticles more uniform. The solution was continued to stir at 37 °C to volatilize tetrahydrofuran (CAT‐NP). C‐NP (CPT‐S‐PEG 12 mg), CA‐NP (CPT‐S‐PEG 12 mg, AZD4635 0.1 mg), CT‐NP (CPT‐S‐PEG 12 mg, TST 0.3 mg), and DSPE‐NP (DSPE‐PEG 12 mg, AZD4635 0.1 mg, TST 0.3 mg) were prepared by the same method. The nanoparticles sizes and zeta potentials were measured by Zetasizer Nano ZS90 (Malvern, UK). All samples were diluted 20‐fold in water before analysis and measured at 25 °C. Morphology of liposomes observed by TEM (JEOL JEM‐1400, Japan) at an operating voltage of 80 kV.

### Characterization the Photothermal and NIR‐II Performance of Co‐Assembly Nanoparticles

TST‐Sol was tetrahydrofuran solution of TST (0.3 mg mL^−1^). Then the water, TST‐Sol, DSPE‐NP, and CAT‐NP were irradiated by 808 nm laser and recorded by infrared thermal imaging camera. Similarly, temperature changes at different concentrations of CAT‐NP or different powers of laser for continuous 5 min irradiation were also recorded. Excited by 808 nm laser with the same power, NIR‐II fluorescence emitted at 900–1500 nm was scanned and NIR imaging was taken. The UV–vis–NIR absorption spectra of TST‐Sol (30 µg mL^−1^) was measured by UH‐4150 ultraviolet‐visible spectrophotometer (Hitachi, Japan). C‐NP was shaken at 37 °C in 1 or 10 mm H_2_O_2_ medium respectively, and samples were taken at different time points for high performance liquid chromatography (HPLC) detection and analysis.

### In Vitro Antitumor Efficacy and Cellular Uptake of TST Nanoparticles

The 4T1 cells were seeded in 96‐well plate with 3000 cells per well and cultured overnight. The old cell culture medium was discarded and fresh cell culture medium containing different nanoparticles was added. The non‐laser group was kept in cell incubator for 24 h, whereas the laser irradiation group was irradiated with 808 nm laser at 1 W cm^−2^ for 5 min after 12 h of dose, and then cultured in cell incubator for another 12 h. Then, MTT was added to each group and incubated for a period of time. The absorbance of each well at 570 nm was detected by microplate reader (Tecan M1000pro, Austria) and the cell viability was calculated. DSPE‐NP and CPT‐NP with lipophilic Cy5.5 (250 µg mL^−1^) were prepared by nanoprecipitation method. Free Cy5.5 was aqueous solution of Cy5.5. The 4T1 cells were seeded in 12‐well plate with 300 000 cells per well. After overnight culture, Free Cy5.5, DSPE‐NP, and CPT‐NP were added to each well and the final concentration of Cy5.5 was 25 µg mL^−1^. After being placed in the cell incubator for 6 h, the cells were detected by flow cytometry (BD FACSCanto‐II, USA) or stained with the nucleus and cytoskeleton respectively by adding Hoechst 33 342 and FITC‐phalloidine, then photographed by confocal laser scanning microscopy (Zeiss LSM880, Germany). The cells were incubated with different nanoparticles for 12 h, then irradiated with 808 nm laser at 1 W cm^−2^ for 5 min, and placed in cell incubator for further culture for 12 h. The cells were scraped off with a cell scraper and RIPA lysis buffer was added to extract proteins. After that, Western blotting experiments were performed to analyze the expression levels of specific proteins. The 4T1 cells were co‐incubated with different nanoparticles for 12 h, and then irradiated with 808 nm laser at 1 W cm^−2^ for 5 min. After that, the cells were further cultured for 12 h, and detected by flow cytometry after Annexin V‐FITC and PI double staining.

### In Vivo NIR‐II, Photothermal and Photoacoustic Imaging

All animal studies were carried out under Institutional Animal Care and Use Committee‐approved protocols of Southern Medical University (202 010 119). The animal model was established by subcutaneously inoculated 4T1 cells in the hind leg of female Balb/c‐nu mice. When the tumor grew to about 200 mm^3^, imaging experiments were conducted. TST‐Sol was first dissolved in *N*,*N*‐dimethylformamide (DMF) to make the concentrated solution, and then diluted with normal saline to obtain 0.3 mg mL^−1^ TST saline solution containing 5% DMF. The concentration of TST in DSPE‐NP and CAT‐NP was 0.3 mg mL^−1^, which was the same as the above‐mentioned preparation method of nanoparticles. NIR‐II imaging: Each mouse was injected with 200 µL of the above solution by tail vein respectively, and NIR‐II fluorescence imaging was performed at 0, 2, 4, 8, 12, 24 h via In vivo Master small animal NIR‐II bioimaging system (Wuhan Grand‐imaging Technology Co., Ltd). Photoacoustic imaging: Each mouse was injected with 200 µL of the above solution by tail vein respectively, and the tumors were photographed with photoacoustic imaging system at 12 h (Vevo LAZR‐X, FUJIFILM, Japan). Photothermal imaging: The 4T1 cells was subcutaneously inoculated in the hind leg of female Balb/c mice. After 12 h of intravenous administration, the tumor sites of mice were irradiated by 808 nm laser at 1 W cm^−2^ power for 2 min and recorded by infrared thermal imaging camera.

### RNA Sequencing Analysis

The 4T1 cells (1 × 10^6^) were seeded in six‐well plates and cultured for 24 h. Then different nanoparticles were added and incubated with cells for 12 h, and irradiated with 808 nm laser at 1 W cm^−2^ for 5 min. Trizol was added to extract RNA after continued culture for another 12 h. BGISEQ‐500 was applied in RNA sequencing. RSEM was used for transcription levels quantification. When the *Q* value was ≤0.001 and the fold change was ≥2, a differentially expressed gene (DEG) was identified. Ggplot2 was employed to generate the volcano graphs. Pheatmap was employed to generate the heat maps. Phyper was utilized to complete Kyoto encyclopedia of genes and genomes (KEGG) pathway enrichment analysis. When the q‐values were ≤0.05, a significant enrichment was identified. Cytoscape was used for the protein–protein interaction network generation.

### In Vivo Antitumor Efficacy Investigation

4T1‐Luc cells were inoculated subcutaneously under the armpit of female Balb/c mice. When the tumor volume reached 100 mm^3^, the nanoparticles were injected into the tail vein to start treatment (TST 3 mg kg^−1^). Twelve hours after intravenous injection, the tumor site was irradiated with 808 nm laser at 1 W cm^−2^ power for 2 min. The medication was given every other day for a total of five times throughout the treatment cycle. The tumor size and body weight were measured daily. When the mice natural death or the tumor size of the mice reached 1500 mm^3^, they were considered dead and recorded. The mice were intraperitoneally injected with d‐luciferin sodium salt (150 mg kg^−1^) on 0, 4, 8, 12 days, and the tumor areas were observed by bioluminescence mode of small animal imaging system (PerkinElmer IVIS Lumina II, USA). On day 12, some mice were sacrificed, and their heart, liver, spleen, lung, kidney, and tumor were dissected for H&E staining pathological sections, then tumor sections were additionally stained with Ki67 and TUNEL. The remaining mice were kept for 30 days and their survival curves were recorded. The tumor tissue was cut into small pieces, then collagenase I was added, and incubated at 37 °C for 1 h. After that, the solution was centrifuged at 1000 rpm for 5 min. The supernatant was collected in the tube, and the cell precipitate was washed twice with PBS and then added into the cell culture medium to obtain single cell suspension. ATP and adenosine contents in supernatant were measured by ELISA. CTL, MDSC, and NK cells were detected via flow cytometry by labeling different antibodies in single cell suspension.

### Statistical Analysis

All the quantitative data were described using the mean SD (standard deviations), and statistical analysis was performed with Student's *t*‐test and one‐way ANOVA via GraphPad Prism 8.0. *p* < 0.05 was considered statistically significant (*p < 0.05).

## Conflict of Interest

The authors declare no conflict of interest.

## Supporting information

Supporting Information

## Data Availability

Research data are not shared.
