# Supplementary material for: Dynamic Adjust of Non‐Radiative and Radiative Attenuation of AIE Molecules Reinforces NIR‐II Imaging Mediated Photothermal Therapy and Immunotherapy
Source: Adv Sci (Weinh). 2022 Jan 22;9(8):2104793. doi: 10.1002/advs.202104793 (PMC8922098; doi:10.1002/advs.202104793)
Supplement: Supplementary file 1 — Supporting Information [file ADVS-9-2104793-s001.pdf]

ADVANCED SCIENCE
Open Access

Supporting Information

for *Adv. Sci.*, DOI 10.1002/advs.202104793

Dynamic Adjust of Non-Radiative and Radiative Attenuation of AIE Molecules Reinforces NIR-II Imaging Mediated Photothermal Therapy and Immunotherapy

Zhenjie Wang, Ling Yu, Yuehua Wang, Chenlu Wang, Qingchun Mu, Xiaojing Liu, Meng Yu, Kang-Nan Wang, Guangyu Yao* and Zhiqiang Yu**

ADVANCED SCIENCE
Open Access

Supporting Information

for *Adv. Sci.*, DOI: 10.1002/advs.202104793

Dynamic Adjust of Non-radiative and Radiative Attenuation of AIE Molecules Reinforces NIR-II Imaging Mediated Photothermal Therapy and Immunotherapy

Zhenjie Wang, Ling Yu, Yuehua Wang, Chenlu Wang, Qingchun Mu, Xiaojing Liu, Meng Yu, Kang-Nan Wang, Guangyu Yao,* and Zhiqiang Yu**

Supporting Information

Dynamic Adjust of Non-radiative and Radiative Attenuation of AIE Molecules Reinforces NIR-II Imaging Mediated Photothermal Therapy and Immunotherapy

Zhenjie Wang,[#] Ling Yu,[#] Yuehua Wang, Chenlu Wang, Qingchun Mu, Xiaojing Liu, Meng Yu, Kang-Nan Wang,* Guangyu Yao,* Zhiqiang Yu*

Dr. Z. Wang, Q. Mu, X. Liu, Prof. Z. Yu

The People's Hospital of Gaozhou

Maoming 525200, P. R. China

Dr. L. Yu

Second Clinical College, Guangzhou University of Chinese Medicine

Guangzhou, P. R. China

AMI Key laboratory of Chinese Medicine in Guangzhou, Guangdong Provincial Hospital of Chinese Medicine,

Guangzhou, P. R. China

Dr. Y. Wang

Cancer Center, Integrated Hospital of Traditional Chinese Medicine, Southern Medical University,

Guangzhou, 510315, P. R. China

C. Wang

MOE Key Laboratory for Analytical Science of Food Safety and Biology College of Chemistry

Fuzhou University,

Fuzhou 350108, P. R. China

Prof. M. Yu, Z. Yu

Guangdong Provincial Key Laboratory of New Drug Screening, School of Pharmaceutical

Sciences, Southern Medical University,
No. 1023, South Shatai Road, Guangzhou 510515, China
Dr. K. Wang
Shunde Hospital, Southern Medical University (The First People's Hospital of Shunde),
Foshan 528308, P. R. China
Dr. G. Yao
Breast Center, Department of General Surgery, Nanfang Hospital, Southern Medical University, Guangzhou, P. R. China，
* Corresponding author
# Contribute equally to this work

1. Materials and methods

1.1 Materials

CPT was obtained from Meryer Co., Ltd., 3,3'-Thiodipropionic acid, 1-ethyl-3(3-dimethylpropylamine) carbodiimide (EDCI) and 4-Dimethylaminopyridine (DMAP) were purchased from Macklin Co., Ltd., Methoxypolyethylene glycol (PEG2000) was obtained from Aladdin Biochemical Technology Co., Ltd. DSPE-PEG2000 was purchased from AVT (Shanghai) Pharmaceutical Tech Co., Ltd.

1.2 Synthesis of TST

TST was synthesized according to the literature.[1-3] Firstly, to a solution of 4,8-dibromo-4H,8H-benzo[1,2-c:4,5-c']bis([1,2,5]thiadiazole) (1.76 g, 5 mmol) and 4-(2-ethylhexyl)-5-(tributylstannyl)thiophen (6.07 g, 12.5 mmol) in freshly distilled THF (50 mL) was bubbled with argon for 20 min. Pd(PPh3)4 (289 mg, 0.25 mmol) was added to the above reaction mixture under an argon atmosphere. The mixture was heated to reflux and it was stirred for 22 h. After cooling, saturated aqueous potassium fluoride (60 mL) was added and stirred at room temperature for 2 h. The reaction mixture was filtered through a Celite pad, the filter cake was washed with dichloromethane. The organic layer was washed with saturated aqueous brine,

dried over anhydrous magnesium sulfate, and the solvent was concentrated under reduced pressure. The crude product was recrystallized from ethyl acetate. The desired product S1 was obtained.

Compound S1 (1168 mg, 2 mmol) was dissolved in a mixture of DMF (20 mL) and MeCN (10 mL). The solution was heated to 65°C and NBS (N-bromosuccinimide) (783 mg, 4.4 mmol) was added in one portion and the reaction stirred in the dark. Then HBr (four drops, 48 weight % aqueous) was added to the reaction. After 10 h, another portion of N-bromosuccinimide (480 mg, 2.7 mmol) was added and the reaction was allowed to continue for an additional10 h. After cooling, the reaction mixture was acidified with hydrochloric acid (180 mL, 2 M) and stirred at room temperature for 2 h. The precipitate was collected by filtration and washed with water and methanol to give the desired compound S3 as an orange-red powder.

To a solution of compound S2 (74 mg, 0.1 mmol) and S3 (67.4 mg, 0.21 mmol) in THF (10 mL) was bubbled with argon for 10 min. Potassium carbonate (36 mg, 0.25 mmol) in1 mL distilled water and Pd(PPh3)4 (23.1 mg, 0.02 mmol) were added to the above reaction mixture under an argon atmosphere. The mixture was heated in an oil bath at 75 °C for 22 h. After cooling to room temperature, the solvent was removed in vacuo. The residue was dissolved in dichloromethane, and the resulting solution was washed with water, saturated aqueous brine. After drying over anhydrous magnesium sulfate and removal of the solvents under reduced pressure. The residue was purified by flash column chromatography on silica gel (10:1 petroleum ether: ethyl acetate to 100 :1 dichloromethane: methanol) to yield the product TST as a dark green solid.

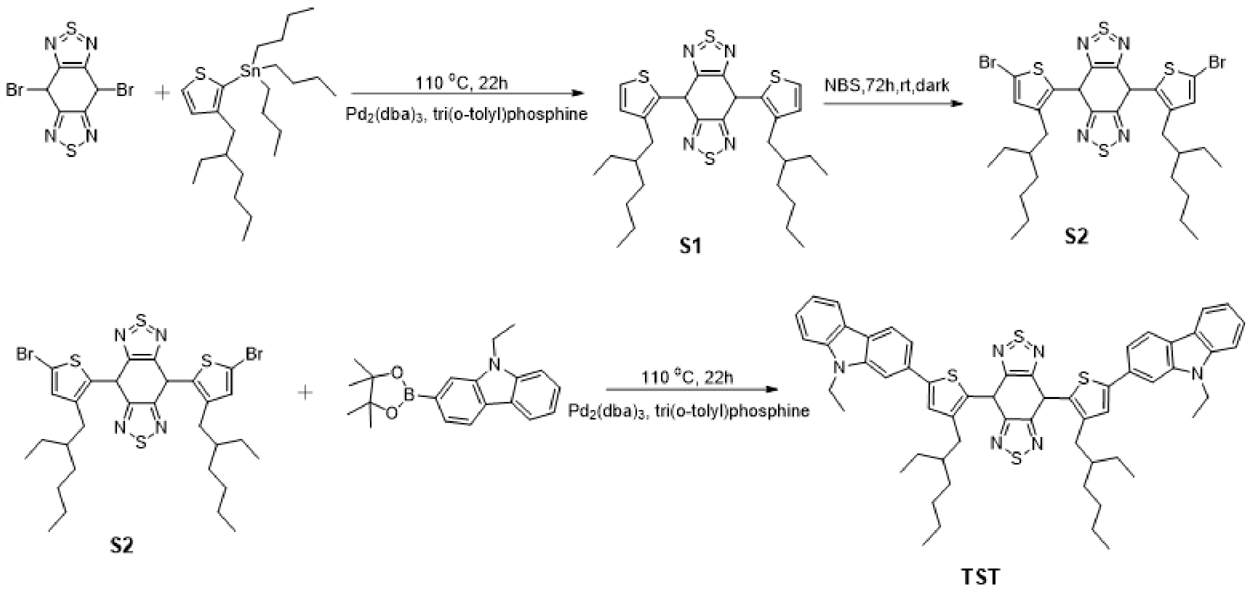

Figure S1. Synthetic routes of TST

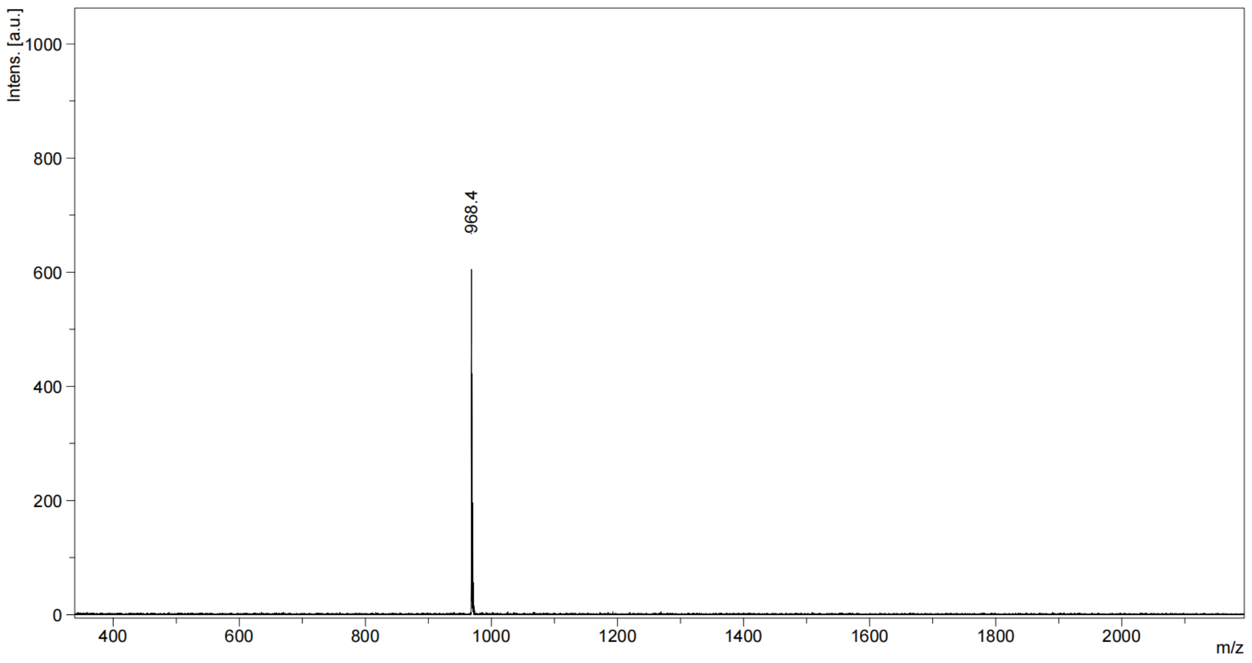

Figure S2. MALDI-TOF-MS of TST ($[M]^+$ 968.4)

^{1}H NMR (400 MHz, $CDCl_3$) δ 8.46 (s, 2H), 8.17 (d, J = 7.7 Hz, 2H), 7.86 (d, J = 8.5 Hz, 2H), 7.49 (d, J = 7.2 Hz, 2H), 7.44 (dd, J = 8.1, 4.3 Hz, 6H), 7.28 (d, J = 7.5 Hz, 2H), 4.41 (q, J = 7.1 Hz, 4H), 2.65 (d, J = 6.8 Hz, 4H), 1.46 (dt, J = 12.5, 6.7 Hz, 8H), 1.20 – 0.81 (m, 16H), 0.67 (t, J = 6.6 Hz, 6H), 0.52 (t, J = 7.3 Hz, 6H).

^{13}C NMR (101 MHz, $CDCl_3$) δ 153.28 (s), 148.29 (s), 144.75 (s), 140.45 (s), 139.79 (s), 128.42

(s), 126.02 (s), 125.45 (d, J = 13.4 Hz), 124.32 (s), 123.41 (s), 122.99 (s), 120.67 (s), 119.16 (s), 118.10 (s), 116.53 (s), 108.73 (d, J = 4.7 Hz), 77.35 (s), 77.03 (s), 76.71 (s), 40.64 (s), 37.73 (s), 34.58 (s), 32.54 (s), 28.64 (s), 25.66 (s), 22.84 (s), 13.98 (d, J = 15.5 Hz), 10.66 (s).

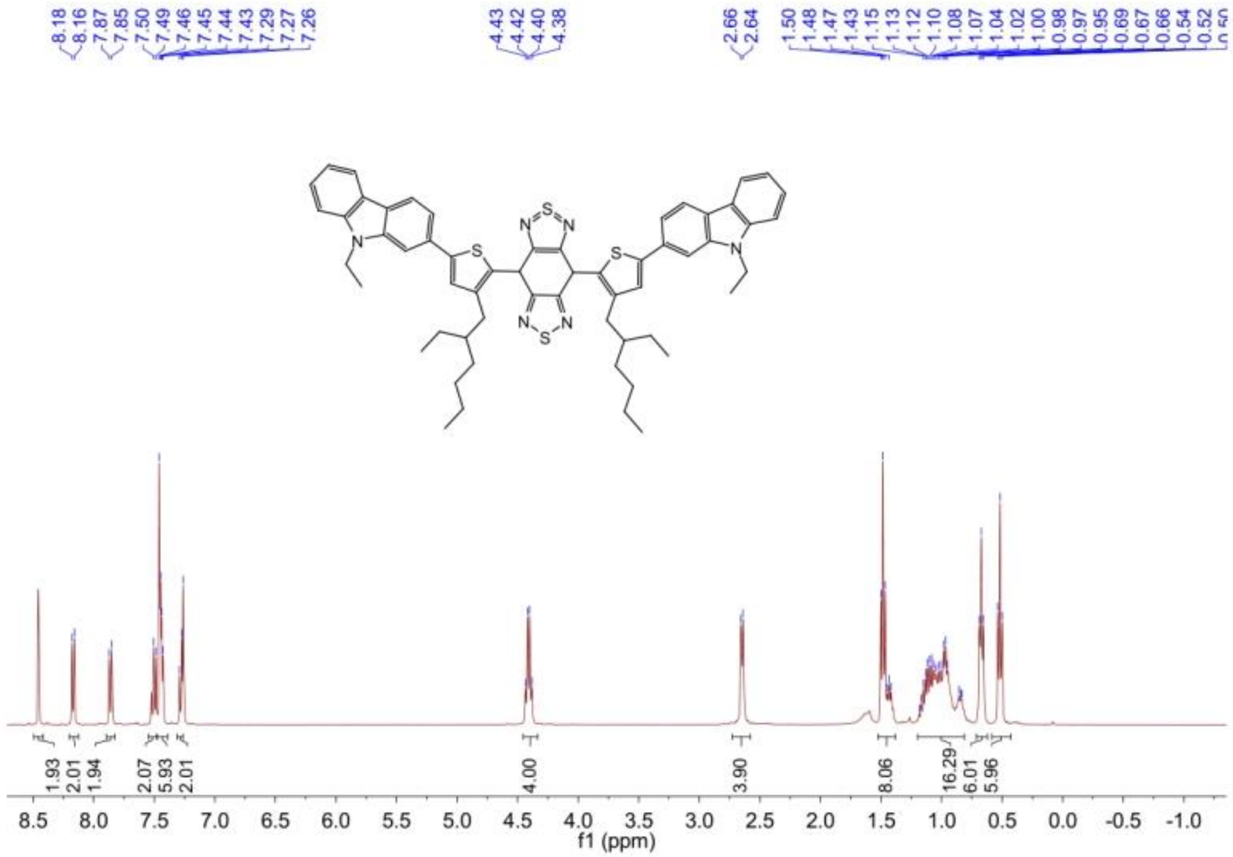

Figure S3. The ^{1}H NMR of TST.

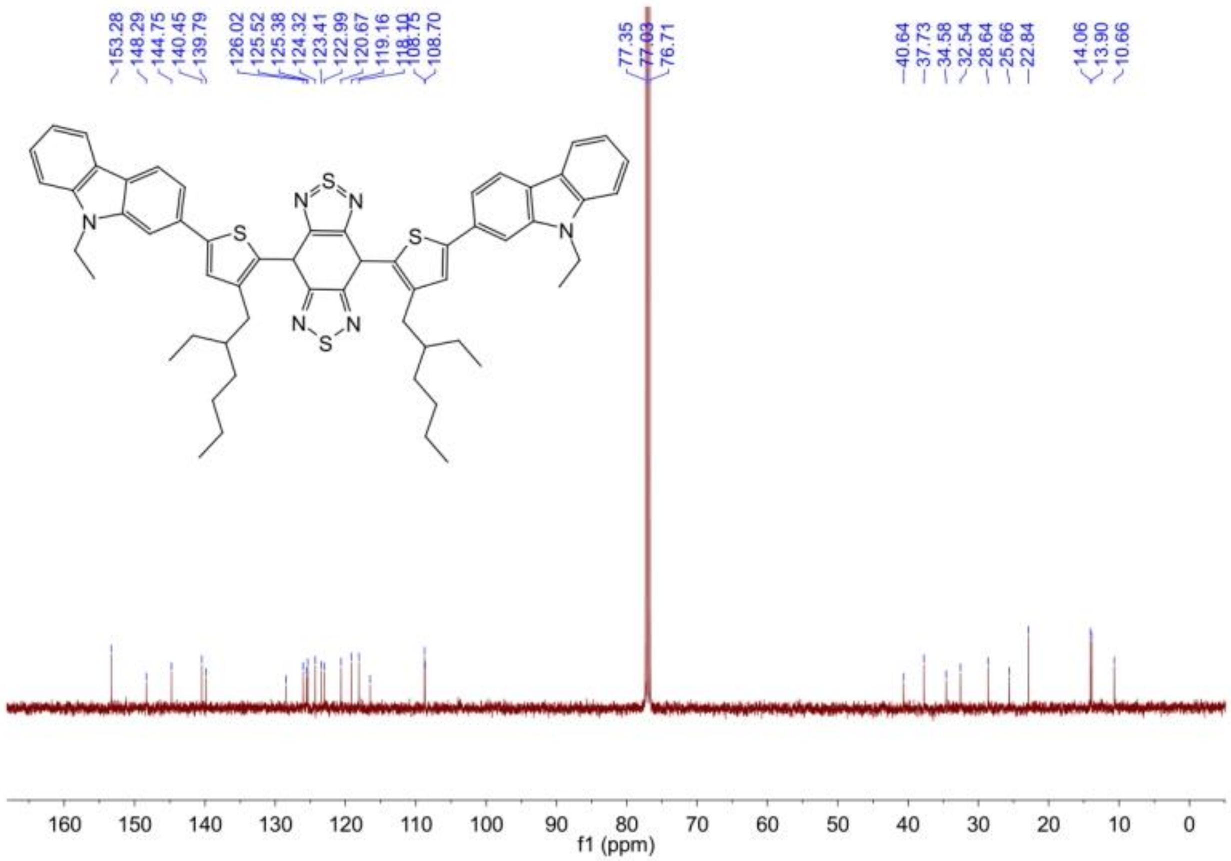

Figure S4. The ^{13}C NMR of TST.

1.3 Synthesis of CPT-S-PEG

First 3,3'-Thiodipropionic acid 1.45 mmol, EDCI 2.88 mmol and DMAP 4.38 mmol were dissolved in anhydrous dichloromethane and stirred at 0 °C for 30 minutes, then CPT 0.73 mmol was added, continuously reacted for 24 h at 37 °C in nitrogen atmosphere. The reaction liquid washed by 1M HCl and saturated sodium chloride solution respectively, and followed by drying the solution with anhydrous sodium sulfate, then purified by silica column chromatography to obtain CPT-S-COOH. And CPT-S-COOH 0.5 mmol, EDCI 1.5 mmol and DMAP 1 mmol were dissolved in anhydrous dichloromethane and stirred at 0 °C for 30 minutes, then PEG2000 1 mmol was added, continuously stirred for 24 h at 37 °C in nitrogen atmosphere. And then purified by silica column chromatography to obtain CPT-S-PEG. The structures of the prodrugs were confirmed by ^{1}H-NMR(Bruker,AV-400,Switzerland) with *d*-DMSO and $CDCl_3$ as solvent.

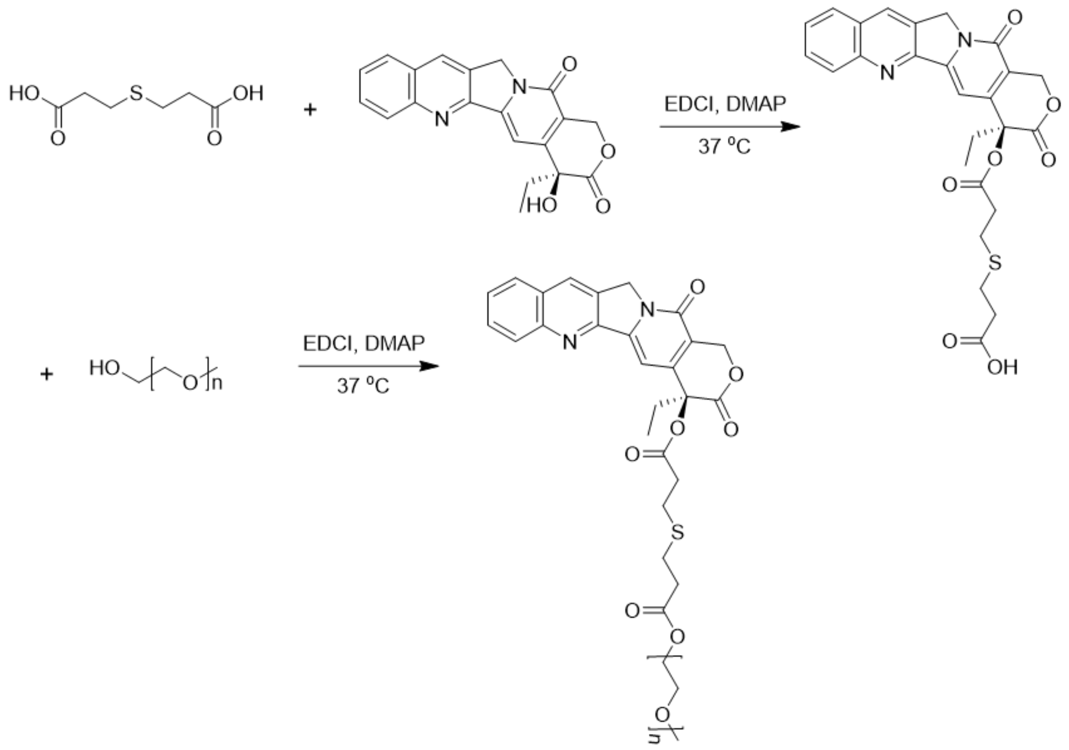

Figure S5. Synthetic routes of CPT-S-PEG

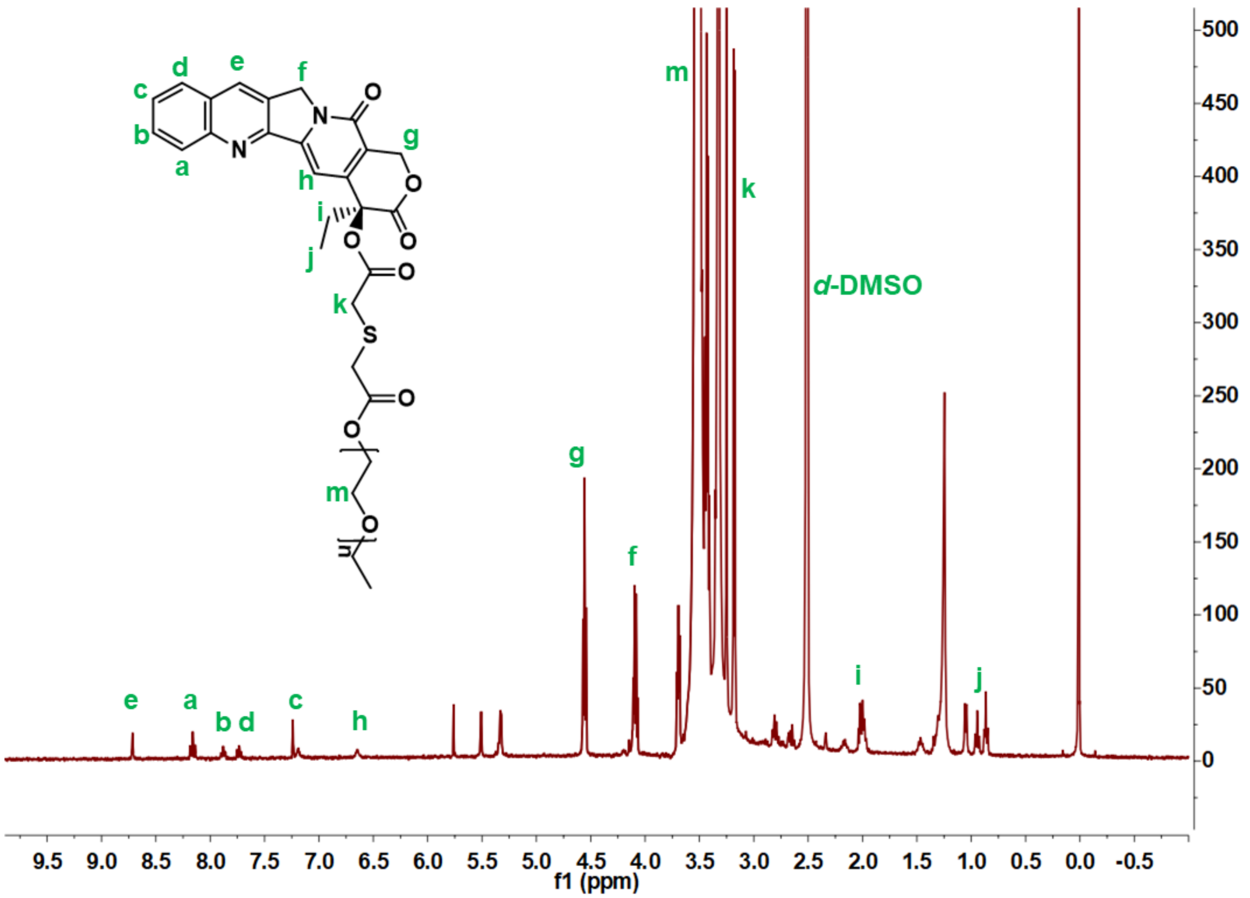

Figure S6. The ^{1}H-NMR of CPT-S-PEG.

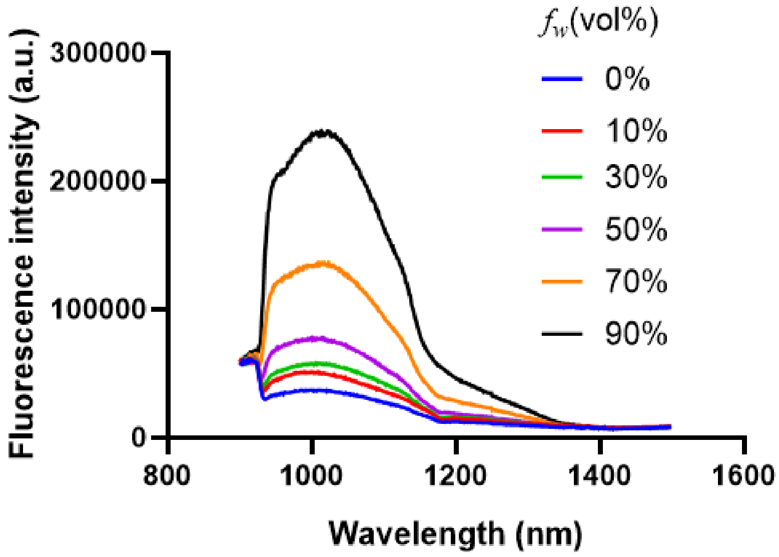

Figure S7. Fluorescence spectra of TST in tetrahydrofuran-H_2O mixtures with different water fractions (f_w).

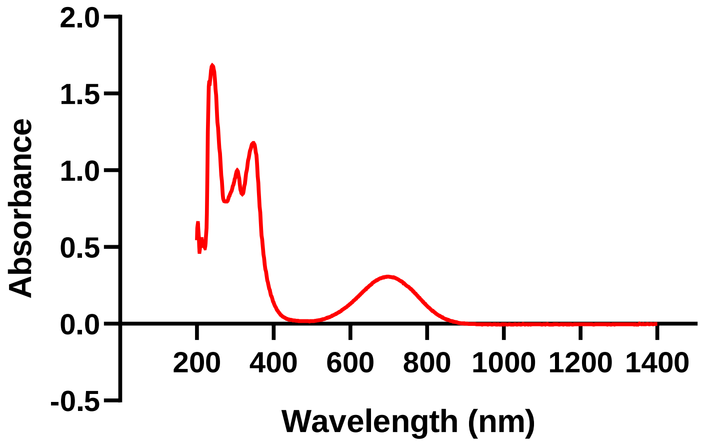

Figure S8. The UV–Vis–NIR absorption spectra of TST-Sol.

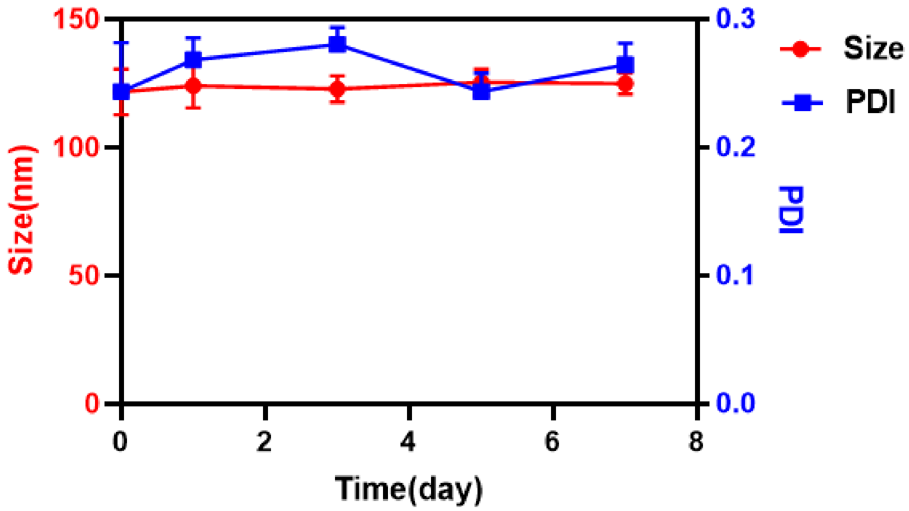

Figure S9. The stability of CAT-NP at 4 ℃ over 7 days.

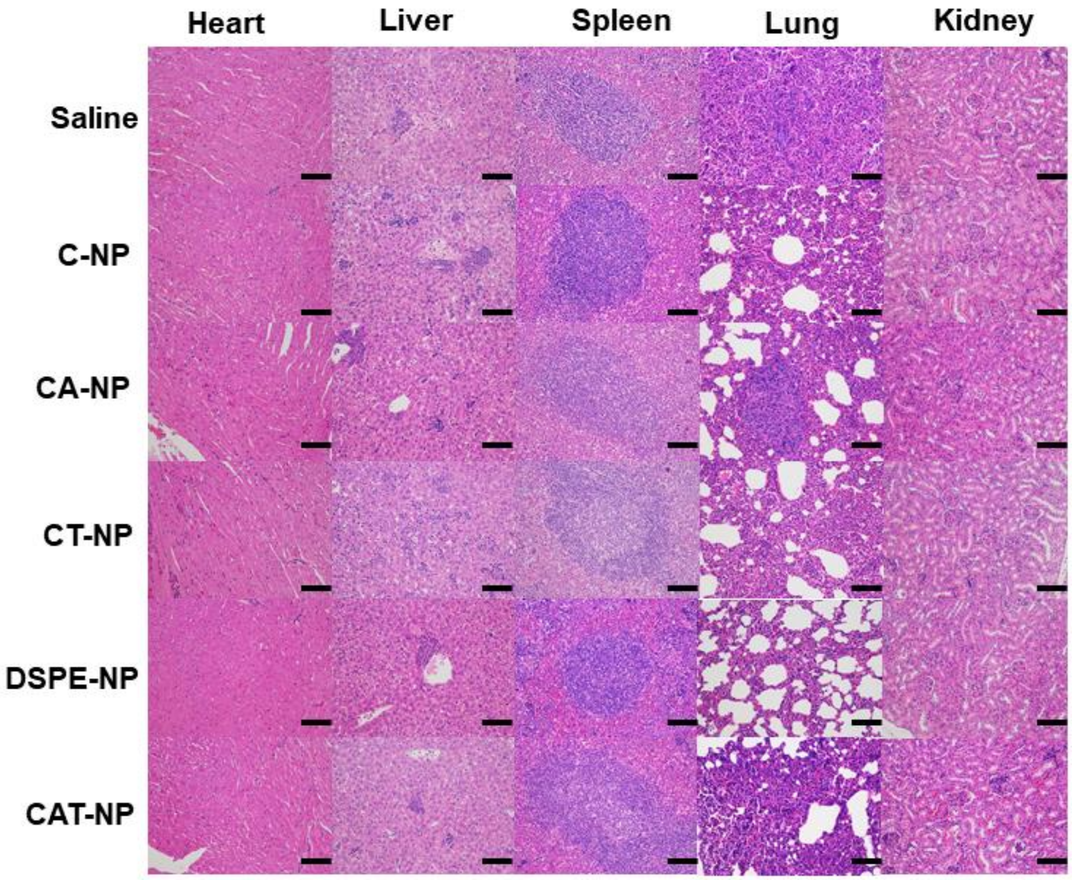

Figure S10. HE staining pathological sections of heart, liver, spleen, lung and kidney after treatment with different nanoparticles, scale bar: 100 μm.

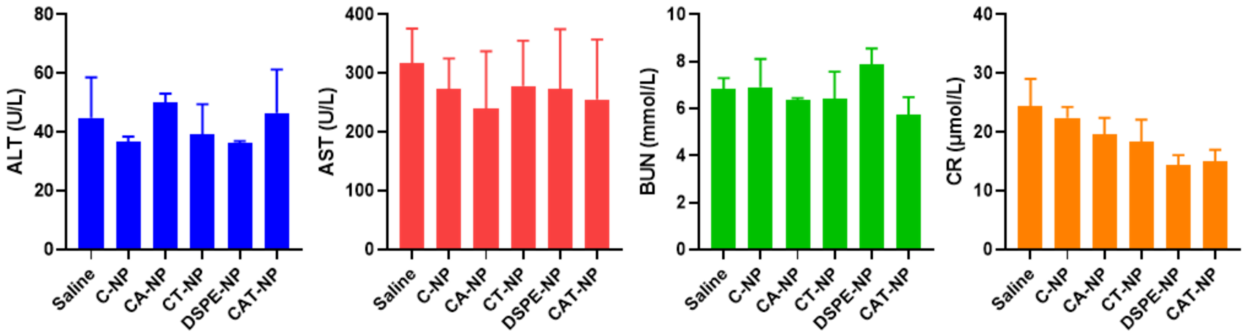

Figure S11. Measurement of biochemical parameters in mice serum after treatment with different nanoparticles. ALT: alanine transaminase, AST: aspartate transaminase, BUN: blood urea nitrogen, CR: creatinine.

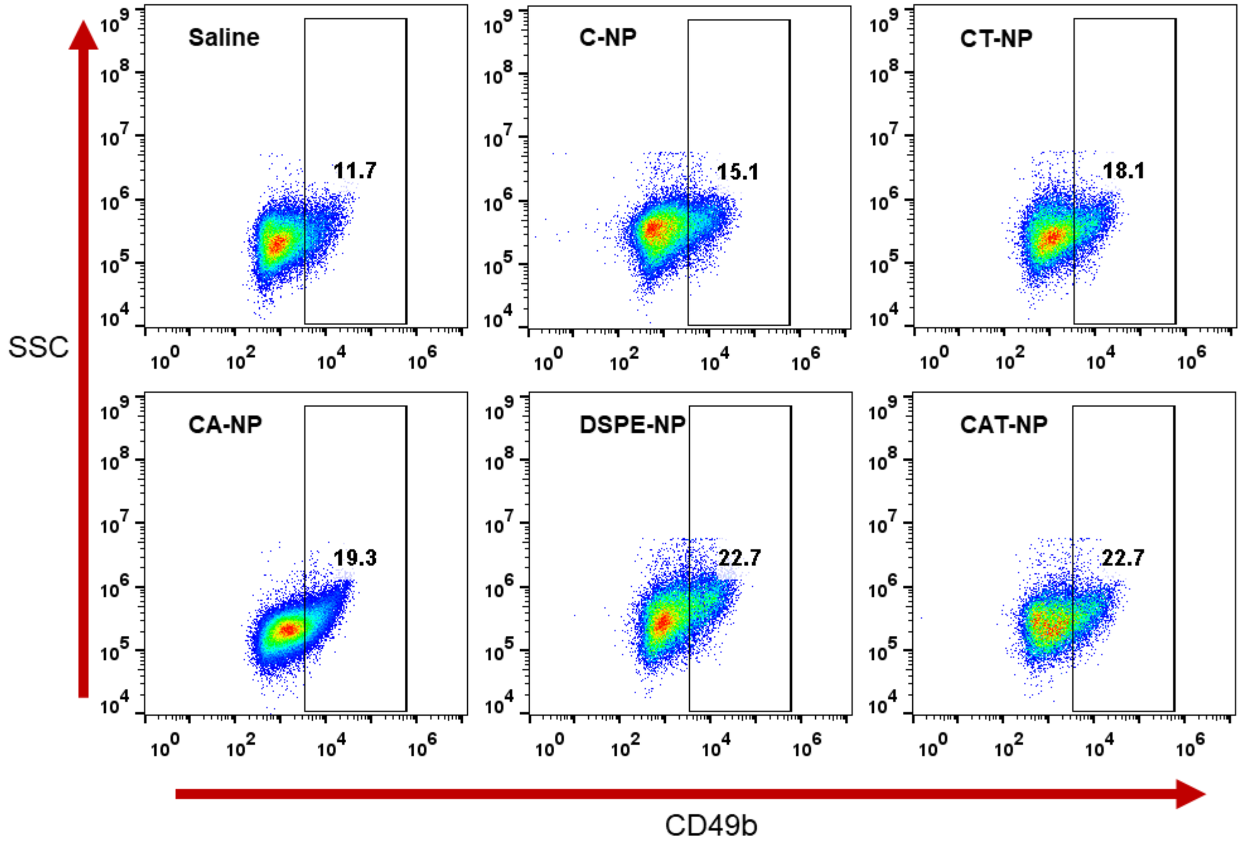

Figure S12. The NK cells in tumor microenvironment after treatment with different nanoparticles.

References:

1. Fang, Y.; Shang, J.; Liu, D.; Shi, W.; Li, X.; Ma, H., Design, Synthesis, and Application of a Small Molecular NIR-II Fluorophore with Maximal Emission beyond 1200 nm. *J Am Chem Soc* **2020,** *142* (36), 15271-15275.

2. Li, J.; Yu, X.; Jiang, Y.; He, S.; Zhang, Y.; Luo, Y.; Pu, K., Second Near-Infrared Photothermal Semiconducting Polymer Nanoadjuvant for Enhanced Cancer Immunotherapy. *Adv Mater* **2020**,

e2003458.

3. Jiang, Y.; Zhao, X.; Huang, J.; Li, J.; Upputuri, P. K.; Sun, H.; Han, X.; Pramanik, M.; Miao, Y.; Duan, H.; Pu, K.; Zhang, R., Transformable hybrid semiconducting polymer nanozyme for second near-infrared photothermal ferrotherapy. *Nat Commun* **2020,** *11* (1), 1857.
